# Using Card Sorting to Explore the Mental Representation of Energy Transition Pathways Among Laypeople

**DOI:** 10.3389/fpsyg.2018.02322

**Published:** 2018-12-04

**Authors:** Rouven Doran, Gisela Böhm, Daniel Hanss

**Affiliations:** ^1^Department of Psychosocial Science, Faculty of Psychology, University of Bergen, Bergen, Norway; ^2^Department of Psychology, Inland Norway University of Applied Sciences, Lillehammer, Norway; ^3^Department of Social Sciences, Hochschule Darmstadt – University of Applied Sciences, Darmstadt, Germany

**Keywords:** mental representation, climate change, energy transition, card sorting, perceived similarity, cross-national, Norway, Germany

## Abstract

Meeting international emission targets will require major changes in the energy system. This paper addresses the public perception of different pathways to energy transition, and their mental representation in particular. A study is reported that employed card sorting to explore how laypeople categorize possible pathway components with respect to their perceived similarity (Norwegian sample, *n* = 61; German sample, *n* = 71). Data sets that were obtained by this method were subjected to multidimensional scaling and cluster analysis. Results for both samples consistently indicate that people differentiate components located at the individual level (e.g., vegetarian food, avoid long flights, walking and cycling), components located at the societal level (e.g., taxes, regulations, urban planning), and components concerned with technological solutions (e.g., hydropower, wind farms, solar panels). These results give reason to assume that laypeople from Norway and Germany share a multifaceted understanding of energy transition, yet some differences between samples were present with regard to the substructure of the individual level category. Future research can build on the present results to explore the subjective meanings of these structures, possibly identifying barriers to public engagement with energy transition.

## Introduction

There is a large scientific consensus that human activities contribute to global climate change, most notably through carbon dioxide emissions (IPCC, 2014). It follows from this scientific insight that the decarbonization of society is paramount in order to meet international targets for limiting global temperature increases (UNFCCC, 2015). One prerequisite for meeting these targets are fundamental changes in energy systems, for instance through increasing the market share of renewables (European Commission, 2011). This paper focuses on exploring the public perception of pathways connected to this energy transition, and on people’s mental representation of such pathways particularly^1^. It has been argued in the psychological literature that studying mental representations can help ensure that interventions designed to implement changes in energy use are communicated and presented in ways that are meaningful for the target audience (Gabe-Thomas et al., 2016).

Böhm et al. (in press) reported findings to suggest that pathways to energy transition are distinguishable according to the level at which they are located: individual actions, societal actions, and technologies. Individual actions are energy-related behaviors performed by individuals, for example at home or at the workplace. Examples are turning down the heat or traveling by public transport rather than by car. Societal actions operate at a larger scale such as through legislation introduced by local or national governments. Typical examples are regulatory policies implemented with the intention to foster low-carbon products and business practices. Technologies refer to the availability and usage of energy sources such as renewables (e.g., hydro, solar, wind). The distinction of these levels has proven useful in several respects. For example, they have emerged as different categories of risks that differ in their public perception and acceptability as well as in the degree of controversy they elicit in public discourse (Fox-Glassman and Weber, 2016; Bassarak et al., 2017). Individual versus collective have also been used to describe levels of action that might differ in their perceived efficacy in tackling problems such as climate change (Lubell, 2002; Koletsou and Mancy, 2011). And preferences for climate action seem to be related to different worldviews such as individualism, egalitarianism or hierarchy (Jones, 2014).

One distinction that has proven fruitful in the psychological literature concerns the type of action that is undertaken, namely curtailment versus efficiency (Gardner and Stern, 2008; Dietz et al., 2009). Curtailment actions reduce energy consumption by cutting back on desired or habitual levels of activity, such as by turning down the heating, usually implying some degree of restriction and limitation of consumption or convenience. Efficiency actions improve the efficiency of energy behaviors without reducing the level of activity and without imposing substantial restrictions. An example are investments in improved housing insulation. Böhm et al. (in press) suggested that this distinction could furthermore prove useful in classifying energy-related actions beyond individuals and households. Examples are transport policies aimed at reducing carbon intensive commuting (e.g., restrictions on inner-city car use, i.e., curtailment action at the societal level), as well as legislation implemented for raising the attractiveness of possible alternatives (e.g., subsidies for electric cars; i.e., efficiency action at the societal level). On a related note, technologies can contribute to making the energy sector less reliant on fossil fuels (e.g., through renewables; i.e., curtailment action at the technological level), whilst other technologies can provide means to cope with carbon emissions that stem from the burning of fossil fuels (e.g., carbon capture and storage; i.e., efficiency action at the technological level).

Baird and Brier (1981) highlighted the role of similarity judgments when laypeople think about energy consumption. Participants were placed in front of paper cards showing a large variety of small-scale (e.g., toaster) and large-scale (e.g., airplane) items. Instructions were to first group the items in whatever manner they wished, and then to rank the items according to their energy requirements. It turned out that the outcome from these two tasks differed in that participants only categorized items alongside their respective energy requirements after explicit instruction. When participants could group the items without explicit instruction, they rather chose to build categories around similarities in function and size. Physical volume was further the dominating feature when participants ranked household appliances based on their anticipated energy consumption per hour. Gabe-Thomas et al. (2016) used a similar method for exploring views about energy consumption among households in another context. Participants received a selection of household appliances with instructions to categorize these appliances according to their similarity and/or dissimilarity. Three separate categories emerged from the participants’ sorting of the appliances. Two of these categories could be interpreted as reflecting a shared meaning, one comprised activities and the other one comprised locations. The appliances in the remaining category shared no dominant theme other than that they were seemingly unrelated to the appliances assigned to the other categories. Other studies have shown that laypeople emphasize curtailment before efficiency when ranking energy devices (or activities) according to their saving potential (Kempton et al., 1985; Attari et al., 2010).

The research presented in this paper utilizes a methodological approach that is reminiscent of the studies conducted by Baird and Brier (1981) and Gabe-Thomas et al. (2016). Rather than directing the participants toward concepts considered relevant by experts, this approach applies card sorting to gain knowledge about how laypeople themselves think about energy issues. Having people judge the similarity between objects is a non-directive way of eliciting mental representations about some issue of interest (Rosenberg and Kim, 1975). This approach leaves it up to the participants how they define similarity and which features of the objects they consider relevant (Barnett, 2008). The general strategy is to derive a structure of the objects from the sorting and then to interpret this structure by trying to identify the underlying criteria that people relied upon throughout the process. The derived structure can be dimensional (yielded by, for example, multidimensional scaling techniques) or categorical (yielded, for example, by cluster analysis). The interpretation of the structure can be enriched by comparing it with data material from relevant reference groups (Canter et al., 1985) and/or by taking into account additional information such as respondents’ knowledge about the concepts under investigation (Barnett, 2008).

In the following, we will report on an empirical study that explored how laypeople think about pathways to change current energy systems into more sustainable ones, for instance by reducing carbon emissions resulting from energy harvesting and use. A card sorting task was used to gain insights into the mental representation of actions, policies, and/or technologies that may contribute to this energy transition. Any single measure to promote change in energy systems will hereafter be referred to as an *energy transition pathway component*^2^. The aim was (i) to investigate how laypeople structure different components in terms of their perceived similarity, and, if possible, (ii) to identify shared patterns underlying these structures. Participants were recruited in Norway and Germany, which are countries shown to differ alongside their current energy profiles (Arnold et al., 2016) and public energy preferences (Steentjes et al., 2017). This allowed us to explore similarities and/or differences in the mental representation of energy transition pathways in a cross-national context.

## Materials and Methods

### Participants

Data were collected between November 2016 and August 2017 at university campuses in Norway and Germany. Participants were invited to take part in the study through e-mail lists, flyers distributed in cafeterias, announcements in classes, and word-of-mouth advertizing. Everybody who took part in the study was offered either a gift voucher worth NOK50 (Norway) or a monetary incentive of €10 (Germany). Each participant was informed about the general aim of the study, that their responses would be anonymous, and that they could withdraw from their participation at any time. Informed consent was assumed through completion of the card sorting task.

In Norway, *n* = 61 students participated in the study, most of which (*n* = 49) were enrolled in the “professional studies in psychology” programme. The remaining participants (*n* = 12) studied work and organizational psychology, comparative politics, constructional and environmental engineering, data technology, design, energy engineering, history, law, product development and production, sociology, or web design. Participants were between 19 and 34 years of age (*M* = 22.97, *SD* = 2.66), and *n* = 48 were female. Only a minor portion of the participants indicated that they had heard the term “energy transition” before (*n* = 16).

In Germany, the sample consisted of *n* = 71 students, thereof *n* = 61 were enrolled in the “business psychology” programme. The remaining participants (*n* = 10) studied information law, social work, or sociology. The age range was from 19 to 53 years (*M* = 24.27, *SD* = 6.82), and *n* = 44 reported to be female. All participants indicated to have heard the term “energy transition” before (*n* = 71).

### Materials

A selection of 25 different energy transition pathway components was presented on paperboard cards, each card featuring one component; an overview is provided in Table 1. The components resemble those used in a study by Böhm et al. (2018) who based their selection upon desk research, interviews with laypeople (i.e., university students), as well as interviews with experts (i.e., climate and political scientists). Each component belonged to one of three implementation levels (i.e., individual actions, societal actions or technologies) and one of two types of energy-related activities (i.e., efficiency or curtailment) described earlier in the introduction.

**Table 1:** List of energy transition pathway components included in the materials.

Label	Energy transition pathway component (translation)	Norwegian sample (original)	German sample (original)
appliances	Energy efficient home appliances (e.g., light bulbs)	Energieffektive husholdningsartikler (f.eks. sparepærer)	Energieeffiziente Haushaltsartikel (z.B. Glühbirnen)
offsets	Climate compensation (e.g., when booking flights)	Klimakvoter	Klimakompensationen (z.B. beim Flüge buchen)
share	Sharing economy (e.g., carpooling)	Delingsøkonomi (f.eks. samkjøring)	Sharing economy (z.B. Fahrgemeinschaften)
vegetarian	Vegetarian food	Vegetarmat	Vegetarisches Essen
no-fly	Avoid long flights	Unngå lange flyreiser	Vermeidung langer Flugreisen
cycle	Walking and cycling	Gå og sykle	Gehen und Rad fahren
political	Political engagement	Politisk engasjement	Politisches Engagement
saving	Energy saving (e.g., turn down heating)	Energisparing (f.eks. skru ned varmen)	Energiesparen (z.B. Heizung herunterdrehen)
science	Science	Vitenskap	Wissenschaft
subsidies	Subsidies (e.g., for renewable energy)	Subsidier (f.eks. for fornybar energi)	Subventionen (z.B. für erneuerbare Energien)
int-agree	International agreements (e.g., on carbon emissions)	Internasjonale avtaler (f.eks. på karbonutslipp)	Internationale Abmachungen (z.B. für Kohlenstoffemissionen)
public-trans	Public transportation	Offentlig transport	Öffentlicher Transport
int-marked	International trade with carbon offsets	Internasjonalt karbonmarked	Internationaler Handel mit Kohlenstoffemissionen
educ	Environmental education (e.g., in school, at work)	Miljøundervisning	Umweltbildung (z.B. in der Schule, bei der Arbeit)
tax	Taxes (e.g., on carbon intensive goods and services)	Skatter (f.eks. på karbonintensive varer og tjenester)	Steuern (z.B. auf kohlenstoffintensive Waren und Dienstleistungen)
regulate	Regulations (e.g., laws to reduce sales of fossil fuel cars)	Reguleringer (f.eks. lover for å redusere salg av fossile biler)	Regulierungen (z.B. Gesetze, um den Verkauf benzin- und dieselbetriebener Autos zu reduzieren)
urban-dev	Urban planning (e.g., car free zones)	Byutvikling (f.eks. bilfri soner)	Stadtplanung (z.B. autofreie Zonen)
nuclear	Nuclear power	Atomkraft	Atomkraft
wind	Wind farms	Vindmølleparker	Windparks
solar	Solar panels	Solcellepaneler	Solarmodule
e-car	Electric cars	Elektriske biler	Elektroautos
water	Hydropower	Vannkraft	Wasserkraft
IT	Information technologies (e.g., monitor home energy use)	Informasjonsteknologier (f.eks. monitorering av energibruk i hjemmet)	Informationstechnologien (z.B. Überwachung des Energieverbrauchs im Haus)
buildings	Energy efficient houses (e.g., geothermal heating)	Energieffektive hus (f.eks. jordvarme)	Energieeffiziente Häuser (z.B. geothermale Wärme)
CCS	Carbon capture and storage	Karbonfangst og -lagring	Kohlenstoffabscheidung und -lagerung

*Paperboard cards concerned with climate compensation [offsets] and environmental education [educ] included an example in parentheses in the German sample but not in the Norwegian sample.*

### Procedure

Participants were invited individually to facilities at the local psychology department. Upon arrival, they were welcomed and seated by a research assistant who introduced the general topic of the study (i.e., the study is about different actions related to energy transition). A definition of the term “energy transition” was provided as well (i.e., long-term changes in energy systems that aim at contributing to a more sustainable society).

The paperboard cards featuring the pathway components were randomly distributed on a table in front of the participants, who were instructed to sort the cards into piles on the basis of perceived similarity. Cards featuring pathway components that were perceived to be similar were to be piled together. Participants were told they should form at least two and a maximum of 25 piles of cards, according to what they considered appropriate. They could leave out cards that they did not want to sort.

After the sorting task, participants were asked what criteria they had used for piling the cards (open response format). The sorting of each participant was documented on a paper form along with the sorting criteria that were mentioned by the participants. Cards that were piled together were assigned the same number. A unique number was used for each pile of cards; the number “0” was assigned to those cards that were not sorted by the participants. The form also provided space for filling in socio-demographic information (i.e., age, gender, and study program), whether participants had heard the term “energy transition” before (yes or no), and possible concluding remarks. Each participant was thanked by the research assistant for taking part in the study and received the voucher or monetary incentive. On average, individual participation took 15 min.

### Analyses

From the sorting that was done by the participants, we derived a measure of similarity of the energy transition pathway components by counting for each pair of components how many participants had placed the pair in one mutual pile and by that had expressed that they considered the two components of the pair similar. Thus, we obtained two similarity matrices of the pathway components, one for the Norwegian and the other for the German sample. The rows as well as the columns of each similarity matrix correspond to the pathway components. Each cell represents a pair of components and contains the number of participants who had placed the pair in a mutual pile. This pairwise similarity measure can range from zero (none of the participants regarded the two components in a pair as similar) to the sample size (all participants regarded the two components in a pair as similar). For technical reasons, the similarities were converted to dissimilarities simply by subtracting the count from the sample size, so that higher numbers now represented greater dissimilarity. This resulted in one dissimilarity matrix for the Norwegian sample and one for the German sample.

The analyses will be reported in the following order: First, we explore the dimensional structure of the dissimilarities by means of a multidimensional scaling analysis (MDS), which represents the empirical dissimilarities as Euclidean distances in a low-dimensional space. This is done separately for the Norwegian and the German data, followed by a discussion of their correspondence. Second, we explore the categorical structure of the dissimilarities by means of a cluster analysis, again analysing the Norwegian and German data separately. Third, we describe an analysis of the open response data provided by participants to report on their subjective criteria employed when completing the sorting task.

All analyses were computed in the R statistical environment (R Core Team, 2018), using the packages smacof, vegan, and Base R for the MDS and cluster analyses, and using the package tm for the analysis of sorting criteria.

## Results

### Dimensional Structure

We conducted non-metric MDS analyses and used the Stress-1 value (Borg and Groenen, 2005) as an indicator of goodness-of-fit. For both the Norwegian and the German sample, we retained the two-dimensional solution, as is indicated by an elbow-like pattern of the stress values across increasing dimensionality of the configuration (similar to a scree test in exploratory factor analysis; cf. Mair et al., 2016). The stress values for the one- to six-dimensional solutions are for the Norwegian sample 0.317, 0.126, 0.075, 0.047, 0.030, and 0.020, respectively, and for the German sample 0.316, 0.120, 0.063, 0.038, 0.028, and 0.019, respectively.

In order to evaluate the goodness-of-fit of the two-dimensional solutions, we conducted a permutation test (500 permutations) as suggested by Mair et al. (2016), which tests the empirical stress value against random permutations of the original data matrix. For the Norwegian sample, the permutation test yielded a mean stress value of 0.31 (σ = 0.01); and a one-sided test with α = 0.5% yielded a critical value of 0.303. For the German sample, mean stress was also 0.31 (σ = 0.01), with a critical value for a one-sided test of 0.301.

Hence, for both the Norwegian and the German sample the observed stress value for the two-dimensional solution was significantly smaller than what would be expected under the null hypothesis of random permutations, indicating a good fit of the configurations to the data. Furthermore, the two-dimensional solutions for both the Norwegian and the German sample proved stable across different starting configurations for the MDS algorithm (Mair et al., 2016). In sum, the two-dimensional configurations can be considered robust and providing good fit to the data.

**FIGURE 1:**
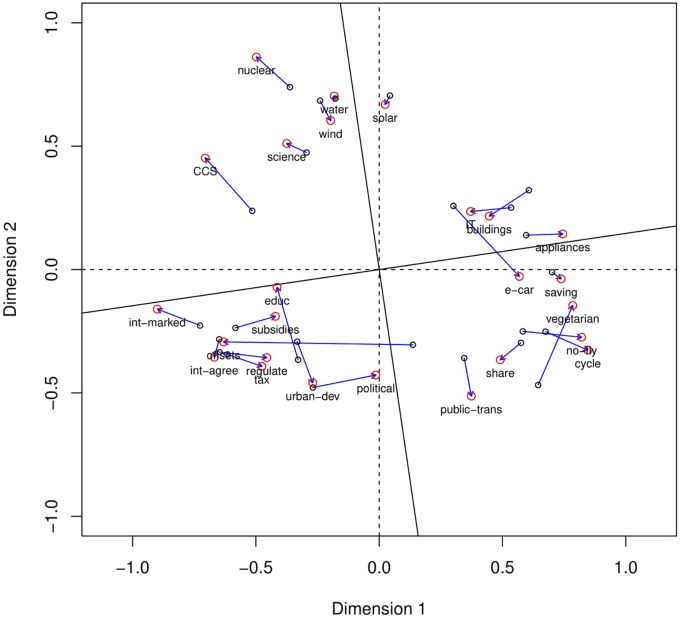
Common plot of the MDS analysis configurations for the Norwegian and the German sorting data after Procrustes rotation of the German configuration. The Norwegian configuration is denoted by red circles, the German configuration by the smaller black circles; arrows indicate the distance between the two locations of an energy transition pathway component in the Norwegian and the German configurations. See Table 1 for the labels of the energy transition pathway components.

The two configurations for the Norwegian and German data turned out to be very similar, which is apparent in their visual appearance (Figure 1) but is also indicated numerically by the correlation of the pairwise distances of the pathway components in the two configurations, *r* = 0.83, *p* = 0.001.

Figure 1 shows the Norwegian and the German configuration in a common plot. We used the Norwegian configuration as the target configuration (denoted by red circles in Figure 1) and the German configuration as the rotated configuration (smaller black circles in Figure 1) in a Procrustes transformation. A Procrustes transformation removes irrelevant differences between two configurations by applying admissible transformations (rotation, dilation, translation) to move one configuration (the rotated configuration) as close to the other (the target configuration) as possible. The two configurations are then directly comparable. The arrows in Figure 1 show how far a pathway component in the German configuration is away from the same component in the Norwegian configuration (labels of the pathway components are placed at the Norwegian configuration).

In both samples, the pathway components form three groups that can be interpreted as corresponding to the presumed three levels (in the following, component labels shown in Figure 1 are given in brackets; see the label column of Table 1 for an explanation of the labels). The distances between the locations of a component in the Norwegian and the German configuration are generally not large in the sense that all components are in the same group in both samples; possibly with the exception of the component climate compensation (offsets) that was placed somewhat closer toward individual actions in the German sample but among the societal actions in the Norwegian sample.

The horizontal axis may reflect a dimension with individual actions to the right (appliances, saving, vegetarian, no-fly, cycle, e-car, share, IT, buildings, public-trans), and societal actions and technologies to the left (CCS, nuclear, science, water, wind, solar, int-marked, int-agree, educ, subsidies, regulate, tax, urban-dev, political)^3^.

The vertical axis may reflect a distinction between ways of implementing behavior change among individuals or groups at the bottom (urban-dev, political, public-trans, int-agree, regulate, tax, share, offsets, no-fly, cycle, int-marked, subsidies, vegetarian, educ, e-car, saving) and technological and engineering solutions at the top (nuclear, water, solar, wind, CCS, science, IT, buildings, appliances).

### Categorical Structure

**FIGURE 2:**
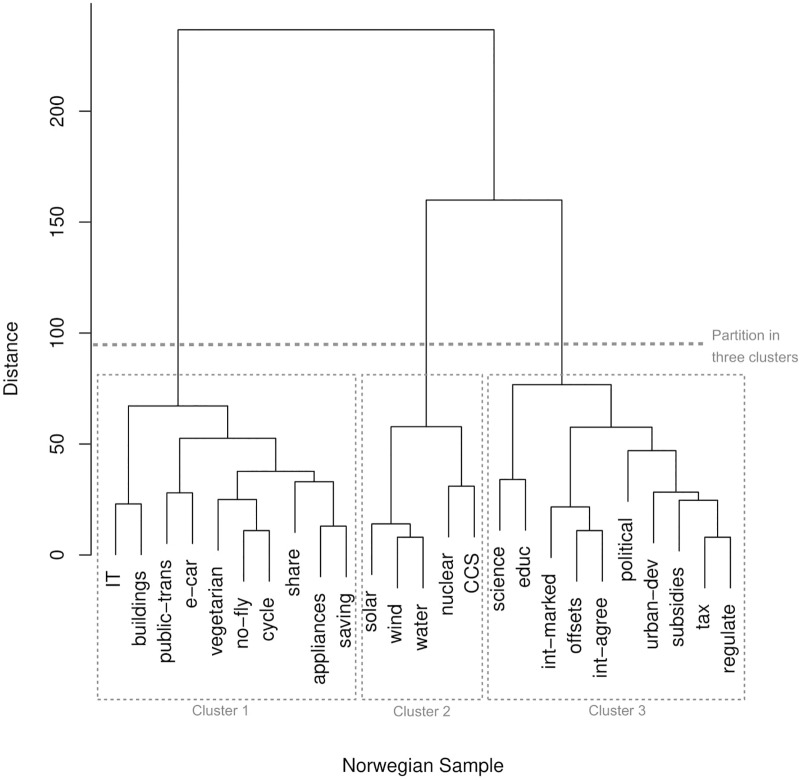
Dendrogram of the hierarchical cluster analysis (Ward) of the Norwegian sorting data. The ordinate axis indicates the distance between merged clusters. Dashed lines indicate the partitioning with three clusters. See Table 1 for the labels of the energy transition pathway components.

**FIGURE 3:**
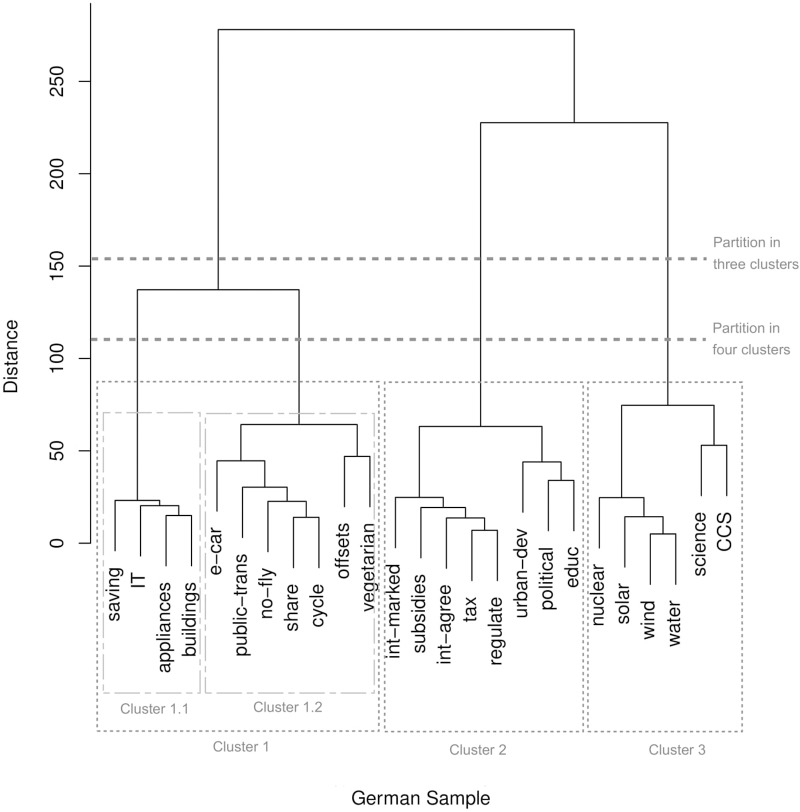
Dendrogram of the hierarchical cluster analysis (Ward) of the German sorting data. The ordinate axis indicates the distance between merged clusters. Dashed lines indicate the partitioning with three and four clusters. See Table 1 for the labels of the energy transition pathway components.

The same dissimilarity matrices that served as input to the MDS analyses were subjected to a hierarchical cluster analysis (Ward method), again separately for the Norwegian and the German data. The dendrograms of the resulting solutions are shown in Figure 2 for the Norwegian sample and in Figure 3 for the German sample. The hierarchical nature of the clustering allows considering classifications with different numbers of clusters, which may reflect varying levels of super- and subordinate mental categorizations.

The cluster solution of the Norwegian data clearly indicates that Norwegian participants categorized the energy transition pathway components into three superordinate categories (in the following, component labels shown in Figures 2, 3 are given in brackets; cf. the label column of Table 1). Cluster 1 makes up pathway components concerned with information technologies (IT), energy efficient houses (buildings), public transportation (public-trans), electric cars (e-car), vegetarian food (vegetarian), flying (no-fly), walking and cycling (cycle), car sharing (share), energy efficient home appliances (appliances) and energy savings (saving). Cluster 2 reflects technologies relating to solar (solar), wind (wind), water (water), nuclear power (nuclear), as well as carbon capture and storage (CCS). Cluster 3 includes policy measures such as science (science), education (educ), international trade (int-marked), climate compensation (offsets), international agreements (int-agree), political engagement (political), urban development (urban-dev), subsidies (subsidies), taxes (tax) and regulations (regulate).

The solution for the German data also indicates three superordinate categories, and the identified structure largely resembles that of the Norwegian data. There are few components whose grouping differed in the two samples. Again, German participants placed offsets together with individual rather than societal actions. Another view at Figure 3 suggests that – at a more subordinate level – individual actions can be divided in two subcategories, namely, pathway components concerned with energy use in the household (saving, IT, appliances, buildings; Cluster 1.1 in Figure 3) and other lifestyle aspects potentially relevant to promote energy transition (e-car, public-trans, no-fly, share, cycle, offsets, vegetarian; Cluster 1.2 in Figure 3).

### Sorting Criteria

The analysis of the sorting criteria focused on term frequencies based on the open response data provided by the participants in both samples. The trimming of the text corpus involved transforming all letters to lower case, removing all numbers and removing punctuation. Further trimming included the removal of stop words (i.e., words that usually do not carry meaning in the respective language) in addition to the stripping of white space (i.e., removal of excessive blanks etc.). This procedure resulted in a total number of *n* = 288 terms in the Norwegian sample, and *n* = 403 terms in the German sample, each of which represented a unique word.

Table 2 lists the thirty most frequently mentioned terms for both samples in order of descending frequency. Many responses involved repetitions of the wording presented on the paperboard cards, yet references about the level at which these pathway components are located were common in both samples. This is evident, for instance, from looking at the words used most often (top ten) when participants elaborated on their sorting criteria. As Table 2 shows, a sizable proportion of these words referred to the level of the component (listed in italics in the following): measure, science, transportation, vegetarian food, nuclear power, *individual level*, climate compensation, level, *public*, and *political* (Norwegian sample); *politics*, science, engagement, *political*, *private*, energy, attributed, do, umbrella term, and possibilities (German sample).

**Table 2:** List of term frequencies for the sorting criteria in each sample.

	Norwegian sample			German sample	
*n*	Terms (original)	Terms (translation)	*n*	Terms (original)	Terms (translation)
42	Tiltak	Measure	25	Politik	Politics
41	Vitenskap	Science	25	Wissenschaft	Science
28	Transport	Transportation	17	Engagement	Engagement
27	Vegetarmat	Vegetarian food	15	Politisches	Political
25	Atomkraft	Nuclear power	15	Privat	Private
21	Individnivå	Individual level	13	Energie	Energy
20	Klimakvoter	Climate compensation	13	Zugeordnet	Attributed
19	Nivå	Level	12	Tun	Do
19	Offentlig	Public	12	Überbegriff	Umbrella term
19	Politisk	Political	11	Möglichkeiten	Possibilities
18	Energi	Energy	10	Staat	State
16	Skatter	Taxes	10	Transport	Transportation
15	Energisparing	Energy saving	8	Energiewende	Energy transition
15	Internasjonalt	International	7	Private	Private
15	Subsidier	Subsidies	6	Ebene	Level
14	Vannkraft	Hydropower	6	Erneuerbare	Renewable
13	Elbiler	E-cars	6	Haushalte	Household
13	Gjøre	Do	6	Internationale	International
13	Kast	Throw	6	Maßnahmen	Measures
12	Byutvikling	Urban planning	6	Überbegriffe	Umbrella terms
12	Hus	House	5	Atomkraft	Nuclear power
12	Internasjonale	International	5	Eigenes	Own
11	Energikilder	Energy sources	5	Energien	Energies
11	Teknologi	Technology	5	Essen	Eating
10	Fornybar	Renewable	5	Haushalt	Household
10	Sykle	Cycling	5	Karten	Cards
9	Biler	Cars	5	Öffentlicher	Public
9	Elektriske	Electric	5	Politische	Political
9	Energieffektive	Energy efficient	5	Subventionen	Subsidies
9	Rest	Rest	5	Vegetarisches	Vegetarian

*Shown are the top-thirty most frequently used terms in descending order. n indicates the term frequency, that is, the number of times that the term was detected in the open response data.*

## Discussion

The present study employed card sorting for tapping into intuitive mental representations about energy transition pathways. The following discussion focuses on two parallel data collections, both asking participants to sort 25 possible pathway components according to their perceived similarity. Results show a close correspondence between the Norwegian and German samples insofar that at least three superordinate categorizations could be distinguished using Ward’s criterion for hierarchical clustering (Figures 2, 3). One cluster can be interpreted as referencing actions concerning individuals and/or households, another cluster seems concerned with technological solutions and the third cluster appears to represent actions located at the societal and/or political level. The overall pattern that emerged from the card sorting fits literature suggesting that laypeople construe energy transition as a multifaceted issue (Böhm et al., in press), but that corresponding mental representations are rather broad (Böhm et al., 2018). This interpretation was supported by the analysis of the open response data in which general terms such as “individual level” or “politics” were frequently used when participants stated criteria based on which they conducted the sorting (Table 2).

Böhm et al. (in press) suggested that possible pathways to energy transition can be distinguished taxonomically based upon their level (i.e., individual, societal, technological) and type (i.e., curtailment, efficiency). The present findings draw parallels to this taxonomy in that participants sorted various components according to the component’s level of implementation. However, the findings do not support the notion that pathway components that concern efficiency (e.g., energy efficient home appliances) are distinguished from those that concern curtailment (e.g., avoid long flights). Apart from showing that different analytical approaches may elicit different mental representations, the proposed distinction in type does not seem to be a readily available concept when laypeople think about energy systems at large. This was unexpected since the proposed distinction emerged in an earlier study exploring impact judgments for some of the pathway components (Böhm et al., in press) addressed in the present study. The finding is also in contrast with studies that have reported empirical evidence to support the distinction between curtailment and efficiency within the context of energy saving behaviors (e.g., Barr et al., 2005; Gardner and Stern, 2008; Karlin et al., 2014; Boudet et al., 2016).

Looking more closely at the results of the hierarchical clustering, there were some differences with respect to the grouping of pathway components focusing on individual actions. In the German sample, participants tended to separate these actions into components related to energy use at home (e.g., energy saving, energy efficient home appliances) and components related to other possible lifestyle choices (e.g., vegetarianism, electric cars, public transportation). This separation corresponds with other studies in which location was identified as a shared theme based on which laypeople categorize behaviors and/or objects related to household water saving (Kneebone et al., 2018) and energy appliances (Gabe-Thomas et al., 2016), amongst others. In the Norwegian sample, in contrast, there was no clear pattern in the data to suggest that Norwegian participants form, similarly, consistent subcategories of individual actions, or of the other two superordinate categories. This difference between the German and the Norwegian sample hints at the direction that German participants’ cognitive structure of energy transition is somewhat more differentiated than that of their Norwegian counterparts. Possibly, this reflects a difference in amount of knowledge about energy transition, as it is known from cognitive psychology that higher expertise in a content domain goes together with finer distinctions; experts use more specific categories than novices (Rosch et al., 1976). Support for assuming that the German sample had more experience with energy transition than the Norwegians did comes from the fact that all German participants but only a small fraction of the Norwegians indicated that they had heard the term energy transition before participating in our study.

The cluster structure emerging from the data closely resembled the spatial patterns obtained in the MDS configurations; both samples yielded three separable regions (Figure 1). An inspection of these configurations suggests at least two dimensions that could possibly underlie the mental representation of energy transition pathways. One dimension seems to indicate varying levels of social aggregation, ranging from pathway components that individuals can implement on their own to pathway components that reflect more of a concerted societal response (cf. horizontal axis in Figure 1). For example, “International agreements (e.g., on carbon emissions)” and “Walking and cycling” were located at opposing ends from another in the spatial structure. Another dimension appears to show different degrees of public involvement, ranging from pathway components that emphasize initiatives to change how individuals and households interact with the energy system to those that comprise technological and engineering solutions to reduce carbon emissions without having to impose substantial restrictions on the everyday activities from individuals and households (cf. vertical axis in Figure 1). For instance, “Nuclear power” and “Urban planning (e.g., car free zones)” were located at opposite locations in the spatial structure.

Research shows that studying meanings ascribed to carbon and energy in everyday contexts can yield insights in public engagement with decarbonization (Whitmarsh et al., 2011). While the present study indicates that certain pathway components are perceived as less similar than others, more data collections are needed to clarify the meanings attached to each one of the identified clusters. One useful addition would be to include materials referencing themes that are prevalent in the public discourse on climate change. Rather than focusing only on single pathway components like renewable energy sources, the sorting may cover more generally phrased paperboard cards such as “climate change mitigation” and “climate change adaption.” This would be informative with respect to the roles laypeople may (or may not) ascribe to themselves in response to climate change, and possible associations with energy use and storage in particular. Another possible extension could be to explore which pathway components are considered most effective with regard to promoting energy transitions. This could be done, for instance, by asking laypeople if they believe that individual actions are less, equally, or more effective in bringing about change in the present energy system than politics and technology. Answering this question would provide insights for researchers and policymakers alike, given that perceived effectiveness in climate mitigation tends to be associated with support for low-carbon policies (Bostrom et al., 2012; Rosentrater et al., 2012).

This study holds several limitations. First, the sorting task was limited to 25 paperboard cards labeled with one energy transition pathway component each. This was done to comply with recommendations in the literature that consider a number between 15 and 25 cards as appropriate in such tasks (Canter et al., 1985). As this selection cannot cover the full range of possible energy transition pathways, interpretations concerning pathways or components not covered in this study must be undertaken with caution. Second, data were collected using single sorting (i.e., without any repetition) rather than multiple sorting (i.e., with one or several repetitions). It is possible that this methodological choice has come at the cost of leaving one or more subordinate categories unidentified, given that multiple sorting tends to be more suitable if the interest is to explore all possible categorization dimensions (Rosenberg and Kim, 1975). Third, the component descriptions on the cards were not entirely consistent across samples. The two cards labeled “Climate compensation (e.g., when booking flights)” and “Environmental education (e.g., in school, at work)” included parenthesized examples in the German sample that were missing in the Norwegian sample. Maybe supplementing pathway component descriptions with an example triggered other interpretations than when no such additional information was provided. For example, this difference might account for the fact that climate compensations were seen closer to the individual actions in the German than in the Norwegian sample. Future studies that employ a similar methodology should try to avoid such inconsistencies to allow for a more unambiguous interpretation of possible sample differences.

## Conclusion

There has been an increasing literature on factors that shape interactions from individuals and households with energy systems (e.g., Stern, 2014). The present paper adds to this literature by shedding light on an aspect that has received relatively little attention, namely on the structures emerging from intuitive categorizations when laypeople think about pathways relevant to energy transition. A study was conducted that employed card sorting to gain insights into the mental representation of possible energy transition pathways in two different countries. Results were consistent in the sense that laypeople structured different pathway components in terms of their respective level of implementation: individual/household, society/politics, or technology. While current initiatives to promote sustainable energy transitions seem to already address pathways at different levels, this study is among the first endeavors to investigate how laypeople mentally represent these pathways and their components. Our results provide new insights also because the allocation of a component to one of the aforementioned levels not always seems obvious. For example, electric cars were grouped together with individual and household actions rather than being allocated to technology. Granted that the findings of this study replicate within the population at large, ideally with representative samples from both countries, this knowledge has potential to improve communication strategies to promote sustainable energy transitions. Including additional measures (e.g., perceived effectiveness) in forthcoming studies could further help identify correlates associated with each super- or subordinate category. This would enable comparisons between different pathway components, and capturing these perceptions would allow systematic comparisons between countries. Policymakers could use this knowledge to identify where public perception matches expert opinion, and if needed, attempt to correct possible misperceptions.

## Data Availability Statement

The raw data supporting the conclusions of this manuscript will be made available by the authors, without undue reservation, to any qualified researcher.

## Ethics Statement

This empirical study complied with the Norwegian Social Science Data Services (NSD) privacy regulations and the ethical principles of research by the National Committee for Research Ethics in the Social Sciences and the Humanities (NESH). Formal approval from NSD was not sought as the collected data material was anonymous, see www.nsd.uib.no/personvernombud/en/notify/index.html.

## Author Contributions

RD and GB contributed conception and design of the study. DH organized and conducted the lab session for the German data collection, and collected the German data. GB performed the statistical analysis. RD wrote the first draft of the manuscript. GB and DH wrote sections of the manuscript. All authors contributed to manuscript revision, read and approved the submitted version.

## Conflict of Interest Statement

The authors declare that the research was conducted in the absence of any commercial or financial relationships that could be construed as a potential conflict of interest.

## Footnotes

1 The term energy transition describes “a change in the state of an energy system as opposed to a change in an individual energy technology or fuel source” (Grubler et al., 2016, p. 18).

2 This is different to an *energy transition pathway*, which can be described as “a combination of steps that are taken with the aim of reducing carbon emissions and improving the sustainability of energy use and production”(Böhm et al., 2018, p. 2).

3 The pathway component with the label offsets was left out of this listing because it was positioned somewhat differently in the two samples.
